# Nanosized Alumina Particle and Proteasome Inhibitor Bortezomib Prevented inflammation and Osteolysis Induced by Titanium Particle via Autophagy and NF-κB Signaling

**DOI:** 10.1038/s41598-020-62254-x

**Published:** 2020-03-27

**Authors:** Zhiwei Zhang, Xuewei Fu, Ling Xu, Xiaolei Hu, Feng Deng, Zhiqiang Yang, Lin Jiang, Tiwei Fu, Pengfei Zhou, Jinlin Song, Ping Ji, Jiao Huang, Xiaomian Wu

**Affiliations:** 1 Stomatological Hospital of Chongqing Medical University, Chongqing Key Laboratory of Oral Diseases and Biomedical Sciences, 401147 Chongqing, P. R. China; 2 Chongqing Medical University, Chongqing Municipal Key Laboratory of Oral Biomedical Engineering of Higher Education, College of Stomatology, 401147 Chongqing, P. R. China; 3 Chongqing Medical University, Department of Orthodontics, College of Stomatology, Chongqing, 40015 P. R. China; 4 Chongqing Medical University, Department of Periodontology, College of Stomatology, Chongqing, 40015 P. R. China; 5 Chongqing Medical University, Department of Prosthodontics, College of Stomatology, Chongqing, 40015 P. R. China; 6 Chongqing Medical University, Key Laboratory of Clinical Laboratory Science, Ministry of Education, College of Laboratory Medicine, Chongqing, 40016 P. R. China; 7 Chongqing Medical University, Department of Oral and Maxillofacial Surgery, College of Stomatology, Chongqing, 401147 P. R. China

**Keywords:** Immunology, Nanoparticles, Biomaterials

## Abstract

Autophagy and NF-κB signaling are involving in the process of Particle Disease, which was caused by the particles released from friction interface of artificial joint, implant materials of particle reinforced composite, scaffolds for tissue engineering, or material for drug delivery. However, the biological interaction of different material particles and the mechanism of proteasome inhibitor, Bortezomib (BTZ), against Titanium (Ti) particle-induced Particle Disease remain unclear. In this study, we evaluated effect of nanosized Alumina (Al) particles and BTZ on reducing and treating the Ti particle-induced inflammatory reaction in MG-63 cells and mouse calvarial osteolysis model. We found that Al particles and BTZ could block apoptosis and NF- κB activation in osteoblasts *in vitro* and in a mouse model of calvarial resorption induced by Ti particles. We found that Al particles and BTZ attenuated the expression of inflammatory cytokines (IL-1β, IL-6, TNF-α). And Al prevented the IL-1β expression induced by Ti via attenuating the NF- κB activation β-TRCP and reducing the expression of Casepase-3. Expressions of autophagy marker LC3 was activated in Ti group, and reduced by Al and/not BTZ. Furthermore, the expressions of OPG were also higher in these groups than the Ti treated group. Collectively, nanosized Al could prevent autophagy and reduce the apoptosis, inflammatory and osteolysis induced by Ti particles. Our data offered a basic data for implant design when it was inevitable to use Ti as biomaterials, considering the outstanding mechanical propertie of Ti. What’s more, proteasome inhibitor BTZ could be a potential therapy for wear particle-induced inflammation and osteogenic activity via regulating the activity of NF- κB signaling pathway.

## Introduction

As a result of rapid advances of the biomaterials science, reliable implants were considered to be most widely accepted operative procedure for treating damaged skeleton and diseased organs, such as various skeletal defects, tooth deficiency, joints replacements and *et al*. Due to the routine use of implants, wear particles or debris can be observed from surfaces in relative friction between implants and physiological environment, surfaces between different component of implant, scaffolds for tissue engineering, or materials for drug delivering^1–4^. In particle disease, peri-implant osteolysis which is caused by wear particles remains the main complication for implant failure after the surgical^5–8^. Although the development of materials (Metal, Polyethylene, Polymethylmethacrylate, and Ceramic) undergone a marvelous evolution over the past half-century, yet none can be considered to be absolutely perfect^9–13^. However, the biological interaction between different types of particles was hardly studied.

In the last 20 years, titanium (Ti) has evolved into one of the most widely used material for implant, tissue engineering, material for drug delivery and composite biomaterials, considered its outstanding mechanical properties^1,13–17^. Nevertheless, Ti implants have some inherent disadvantages. The poor early stability and insufficient stress conduction cannot afford a stabilized bonding between implants and the peripheral bone. Mechanical instability of the bone-implant interface will generate wear particles from any part of the implant^18,19^. Aluminum is one of biomaterials and wildly used for diagnostic, therapeutic material, and replacement for bone defect. It is considered as a low acute cytotoxicity compared with other metal materials^20^. Particle disease is the resultant of wear particles production and progresses with time, whose pathophysiology is still unclear. Researchers are attempting to study its potential mechanism to prevent osteolysis induced by wear particles. It is illuminated that nuclear factor-kB (NF-kB) is an important modulator of particle disease, which plays key roles in the process of osteoblastic inhibition and osteoclastic formation. Considerable evidences suggest that Ti wear particles can induce the inflammatory reaction, while high dose of Ti particles exceeding the toxic level will lead to cells aoptosis and necrosis probably^21–23^. Macrophages, osteoblast, osteoclast, fibroblasts, mesenchymal stem cell (MSC), and T cells will secrete numerous proinflammatory chemokines and cytokines in response to wear particles stimulate, such as interleukin 6 (IL-6), tumor necrosis factor alpha (TNF-α), interleukin 1 beta (IL-1β), prostaglandin E2, and macrophage colony stimulating factor (M-CSF)^5,24–30^. Subsequently, the secretion of various cytokines will stimulate the expression of the receptor activator of NF-kB ligand (RANKL), an essential factor for regulating bone remodeling. Then, RANKL interacts with RANK, which will activate the downstream signaling pathways and further promote proinflammatory chemokines expression^8,29,31^. During the past decade, it is an increasing interest in the relationship between osteoblasts and peri-implant osteolysis. In fact, wear particles are more likely to inhibit bone formation than induce bone resorption around unstable implants^32–35^.

Previous studies have shown that the ubiquitin–proteasome system (UPS) plays an essential role in regulating NF-κB activity^36–38^. Bortezomib (BTZ), which is one of the reversible proteasome inhibitors, has been shown to be a potent inhibitor of NF-κB^38,39^. Bortezomib could block the nuclear transport of NF-kappaB, mainly by inhibiting the chymotryptic activity of polyubiquitinated protein degradation in 26S proteasome^30,40–43^. Bortezomib at the first has been approved by FDA as a drug for the therapy of tumor, but it is now also considered as a remedy for non-tumorous diseases such as inflammation diseases^44–46^. And the dose-dependent manner of Bortezomib is reported by lots of studies. At a low dose, it not only could not induced cell apoptosis but also could protect cell from apoptosis^44^. And our primary showed that bortezomib at a low dose (<0.5 nM) prevented inflammatory reaction of periodontal ligament cells without influencing the cell cycle or cell bioactivity^45^. It is reported that autophagy induces cell migration and proliferation of pulmonary arterial smooth muscle cells via NF-κB pathway. And blocking ROS-NF-κB-dependent autophagy could promote the apoptosis in squamous cell carcinoma^47,48^.

Although the toxicity of Ti particle was proved^5,8,12^, and the Ti particles released from Ti bulk implant or composites was reported by lots of study^1,2,16,22^, it was inevitable to use Ti as an implant material, tissue engineering material, material for drug delivery or particle reinforced composite, considering its outstanding mechanical property^13,14,17^. Therefore, in this study, we are attempting to investigate the potential mechanisms of potential therapy for wear particles-induced osteolysis and explore the biological interaction between Ti and Al-NPs. We aimed to find out the effects of BTZ at a low dose on bone formation and wear particles-induced osteolysis as well investigate the potential mechanisms, considering its effect on inflammation disease and dose-dependent manner. Findings of our study may provide a potential method for preventing wear particle-induced prosthesis loosening in the clinic and a better strategy for implant design^44–46^.

## Results

### The effect of aluminum particles on apoptosis and necrosis of mg-63 cells induced by titanium particle

**Figure 1:**
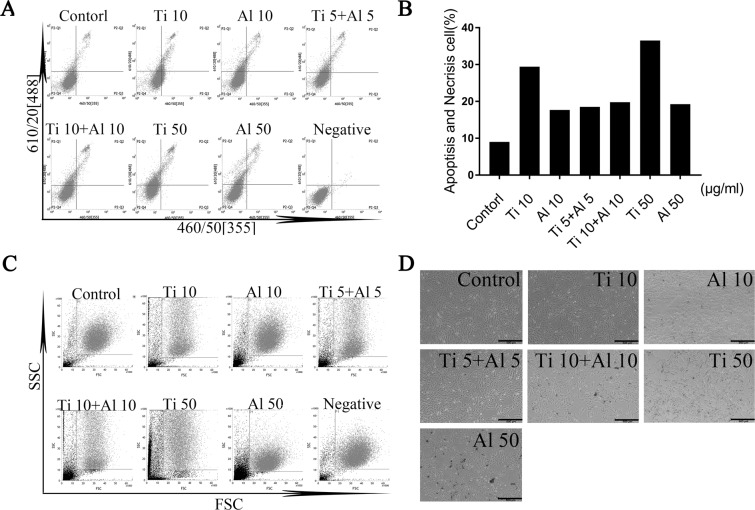
The effect of Al-NPs on the apoptosis and necrosis of MG-63 cells Induced by titanium oxide (Ti) Particle. (**A,B**) Cells toxicity was measured on day 3 after treating with different concentration particles in flow cytometry assay. (**C**) Forward-scattered (FSC)-H light and side scattered (SSC)-H respectively present the cellular size distribution and particle complexity. (**D**) Images of MG-63 cells treated with different concentration of practices for three days with light microscopes (Bar = 100, 500 μm).(Group: Control, Ti 10: 10 µg/ml Ti, Al 10: 10 µg/ml Al-NPs, Ti 5 + Al 5: 5 µg/ml Ti + 5 µg/ml Al-NPs, Ti 10 + Al 10: 10 µg/ml Ti + 10 µg/ml Al-NPs, Ti 50: 50 µg/ml Ti, Al 50: 50 µg/ml Al-NPs).

Apoptosis and Necrosis Assay showed that Ti particles at 10 µg/ml and 50 µg/ml had markedly pro-apoptotic effect. Almost 30% of MG-63 cells were induced apoptosis treated with Ti particles at 50 µg/ml (Fig. 1A,B). However, Al-NPs had no distinct pro-apoptotic effect on MG-63 cells. Moreover, the number of apoptotic MG-63 cells induced by Ti particles was reduced once co-cultured with Al-NPs. In the meantime, the morphology of MG-63 cells was observed with optical microscope (Fig. 1D). The cellular size distribution and particle complexity increased in Ti particles concentration-dependent manner, whereas the cellular size distribution had not obviously changed in Al-NPs groups (Fig. 1C). The results showed that Al-NPs had no distinct pro-apoptotic effect and could efficiently alleviate toxicity of Ti particles. The groups that Ti practices mixing with Al-NPs exhibited lower toxic than Ti alone. And in the same concentration of 10 µg/ml, Ti was more toxic than Al-NPs. Based on these results, in order to minimize the effect of concentration and figure out the effect of Al-NPs on the toxicity of Ti particle, the low concentration of both materials (10 µg/ml Ti, 10 µg/ml Al-NPs) were used in the following experiments.

### Toxicity of BTZ on MG-63 cells

**Figure 2:**
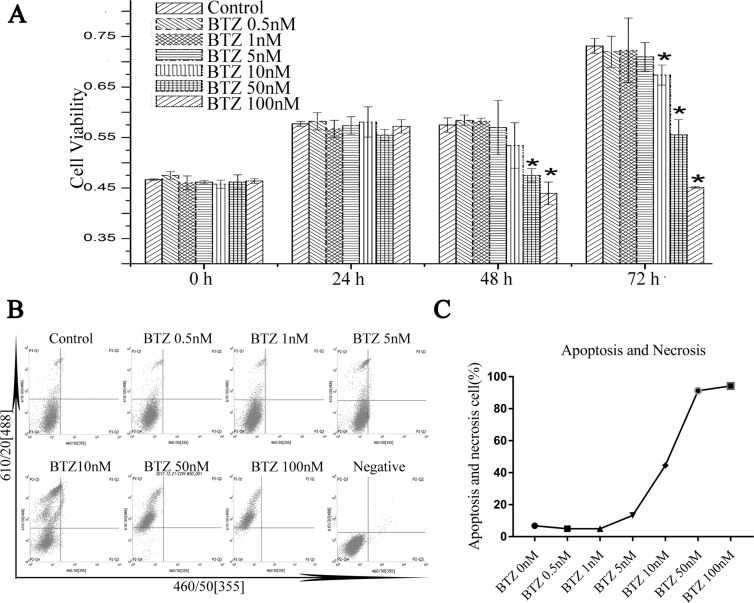
Effects of Bortezomib (BTZ) on the viability and cytotoxicity of MG-63 cells. (**A**) Cells viability was measured on day 0, day 1, day2, day3 after treating with BTZ (0 nM, 0.5 nM, 1 nM, 5 nM, 10 nM, 50 nM, and 100 nM) using MTT assays. The results are expressed as mean ± SD. (**B,C**) After treating with BTZ (0 nM, 0.5 nM, 1,5 nM, 10 nM, 50 nM, and 100 nM), cells were collected on day3 and detected by Apoptosis and Necrosis Assay Kit. *Compared with the control group, p < 0.05.

Our MTT proliferation assay and Apoptosis assay showed that BTZ under the concentration of 1 nM did not affect cell proliferation and had no pro-apoptotic effect on MG 63 over the whole culture duration (Fig. 2A–C and Supplementary Fig. 1). By contrast, the concentration above 10 nM markedly suppressed cell proliferation (Fig. 2A). While the concentration up to 5 nM induced apoptosis of the cells obviously (Fig. 2C). Based on results of above, the non-cytotoxic concentration (BTZ 0.5 nM) was used in subsequent experiments.

### Ubiquitin proteasome inhibitor and Aluminum particles prevented the activation of NF-κB signaling pathway and autophagy induced by titanium particle

**Figure 3:**
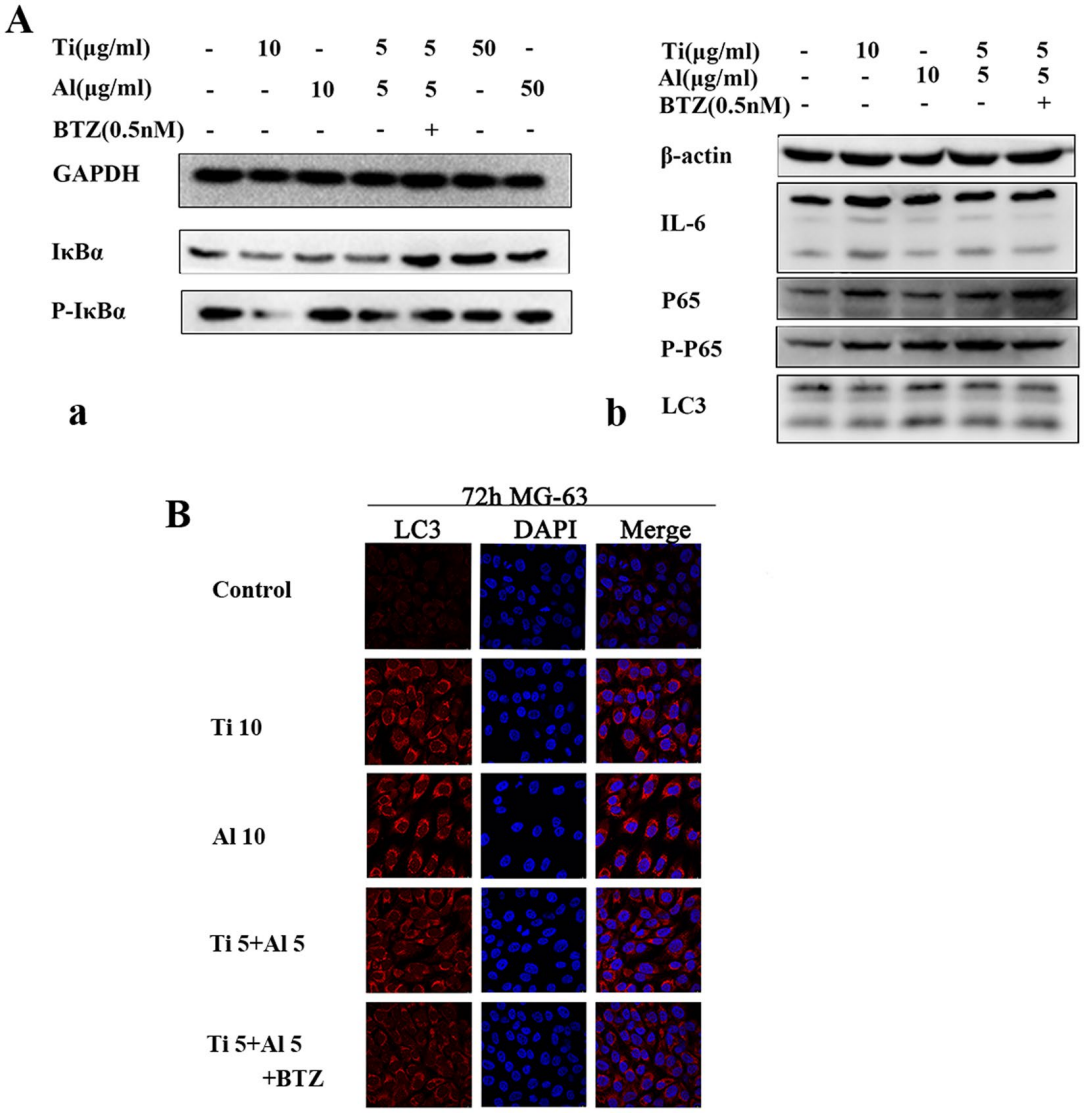
Expression of NF-κB signaling pathway proteins and LC3. (**A**) Expression of NF-κB signaling pathway proteins (IκB, IκB-α, P65, P-P65), LC3 and IL-6 were analyzed using western blot. (**B**) Immunofluorescence staining assay was performed to detect the expression of LC3 (Every antibody of grouping of gels/blots was exposed from the same gel without any cropping). Red fluorescence represented LC3, While Blue fluorescence represented Dapi.

Western blot and immunofluorescence staining assays were performed to elucidate the effect of ubiquitin proteasome inhibitor and Al-NPs on autophagy and activation of NF-κB signaling pathway induced by Ti Particle in MG-63. IκBα is an inhibitor of NF-κB. Western blot analysis showed a lower expression of IκBα and enhanced expression P-IκBα/IκBα in Ti treated group. The protein concentrations of P-IκBα/IκBα slight decreased in Al-NPs and Ti 5 + Al-NPs 5 treated groups compared with Ti treated group (Fig. 3A and Supplementary Figs. 2 and 3) and increased in the Al-NPs group, suggesting that Ti particles activated NF-κB Signaling Pathway, meanwhile, Al-NPs suppressed NF-κB activation by inhibiting IκBα degradation via the accumulation of P-IκBα. The protein concentration of P-IκBα/IκBα and p-p65/p65 was decreased after the cells were treated with BTZ, illuminating that ubiquitin proteasome inhibitor prevented the activation of NF-κB signaling pathway. Furthermore, the expression levels of IL-6, one of the NF-κB downstream gene were increased in Ti treated group, but were lower in Al-NPs treated groups. And the protein concentrations of LC3 was increased in Al-NPs group, and decreased in Ti 5 + Al-NPs 5 and Ti 5 + Al 5 + BTZ groups compared to Ti group. These results indicate that ubiquitin proteasome inhibitor and Al-NPs not only prevented the activation of NF-κB signaling pathway, but also evoked the cell autophagy which could prevent the apoptosis of MG 63 as shown above. Again, in the immunofluorescence staining assays showed that the LC3 was strong expressed in the Ti group, Al-NPs group and Ti 5 + Al-NPs 5, especially in the Al-NPs group, but slightly dropped in the Ti 5 + Al-NPs 5 + BTZ groups (Fig. 3B). All of these indicated that autophogy played an important role in the mechanism that how BTZ alleviated the toxicity of Ti particles on MG63.

### Ubiquitin proteasome inhibitor and aluminum particles prevented the activation of NF-κB signaling pathway by RT-PCR assay

**Figure 4:**
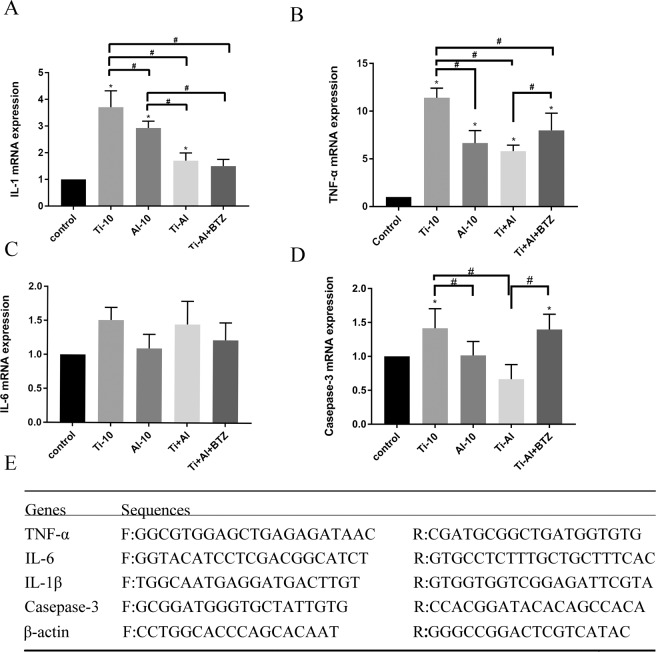
Gene expression of inflammatory and apoptosis related cytokines. (**A–D**) To determine the effects of Al-NPs and BTZ, mRNA expression of IL-1β, IL-6, TNF-α and casepase-3 were determined using real-time PCR. As shown in Fig. 4, RNA expression of these pro-inflammatory cytokines increased after 3 days of Ti-10ug/ml treatment (p < 0.05), but the expression was reduced by co-culturing with Al and/not BTZ (0.5 nM) treatment. *Compared with the control group, p < 0.05; ^#^compared with the Ti group, p < 0.05. (**E**) The primers for PCR experiment.

RT-PCR analysis revealed that Ti particle provoked a significantly higher expression of inflammatory mediators, including IL-1β, IL-6, TNF-α (Fig. 4A–C) and inflammation response was significantly attenuated the by co-cultured Ti particles with Al-NPs, especially when treated with BTZ (p < 0.05). These data suggested that BTZ could downregulate pro-inflammatory cytokines in MG-63 cells induced by Ti particles via regulating NF-κB Signaling Pathway. Meanwhile, Ti particles elevated the expression of caspase-3 gene, while Al-NPs again prevented the expression of caspase-3 gene (Fig. 4D), but BTZ could not significantly blocked the caspase-3 expression in this process (p > 0.05). All these results indicated that besides NF-kB signaling pathway, there should be other signaling pathway modulated the biological interaction between Ti and Al-NPs in MG 63 cells. And the autophogy pathway should be one of them as shown above.

### Al-NPs particle reduced the IL-1β releasing induced by Ti-μ via attenuating the activator of NF-κB signaling β-TRCP

**Figure 5:**
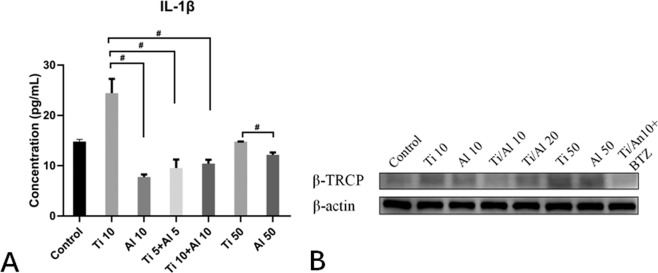
Al-NPs particle reduced the IL-1β releasing induced by Ti-μ via attenuating the activator of NF-κB signaling β-TRCP. (**A**) Al-NPs prevented the Ti particles-induced IL-1β releasing in Enzyme-Linked Immunosorbent Assay (ELISA). Not only in the 10 μg/ml Al group and 5 μg/ml Ti + 5 μg/ml Al group but also in the 10 μg/ml Ti + 10 μg/ml Al group, Al-NPs significantly reduced the IL-1β expression, compared to 10 μg/ml Ti group (^#^p < 0.05). And at both concentration 10 μg/ml and 50 μg/ml, the Al-NPs could induce a higher level of IL-1β expression (^#^p < 0.05). (**B**) Protein expression was determined using Western Blot. Both in the 5 μg/ml Ti + 5 μg/ml Al group and 10 μg/ml Ti + 10 μg/ml Al group, Al-NPs attenuated the activator of NF-κB signaling β-TRCP. (Group: Control, Ti 10: 10 µg/ml Ti, Al 10: 10 µg/ml Al-NPs, Ti 5 + Al 5: 5 µg/ml Ti + 5 µg/ml Al-NPs, Ti 10 + Al 10: 10 µg/ml Ti+ 10 µg/ml Al-NPs, Ti 50: 50 µg/ml Ti, Al 50: 50 µg/ml Al-NPs, Ti 10 + Al 10 + BTZ:10 µg/ml Ti + 10 µg/ml Al-NPs + 0.5 nM BTZ) (^#^p < 0.05).

In order to further study the mechanism of Al-NPs preventing IL-1β expression induced by Ti, we performed the Immunosorbent Assay (ELISA) and western blot experiment. As the Fig. 5 shown, not only in the 10 μg/ml Al group and 5 μg/ml Ti+ 5 μg/ml Al group but also in the 10 μg/ml Ti+ 10 μg/ml Al group, Al-NPs significantly reduced the IL-1β expression, compared to 10 μg/ml Ti group (^#^p < 0.05). The expression of IL-1β significantly increased in Ti 10 ug/m group compared to the control and Al 10 ug/ml group in the ELISA experiment (Fig. 5A) (^#^p < 0.05). And the Al-NPs significantly reduced the IL-1β in the 5 μg/ml Ti+ 5 ug/ml Al group (^#^p < 0.05). What’s more, keeping the same Ti concentration in 10 μg/ml Ti + 10 ug/ml Al group with a total particle concentration of 20 μg/ml, the Al-NPs could reduce the IL-1β expression induced by Ti rather than increasing the expression of IL-1β compared to10 μg/ml Ti group (^#^p < 0.05). And at both concentration 10 μg/ml and 50 μg/ml, the Al-NPs could induce a significant higher level of IL-1β expression (^#^p < 0.05). In the western blot assay (Fig. 5B and Supplementary Fig. 4), we found that both in the 5 μg/ml Ti + 5 μg/ml Al group and 10 μg/ml Ti + 10 μg/ml Al group, Al-NPs attenuated the activator of NF-κB signaling β-TRCP, compared to 10 μg/ml Ti group. And a low dose of BTZ (0.5 nM) could block the β-TRCP expression induced by particles.

### Effects of Al and BTZ on Ti particles-induced calvarial osteolysis model

**Figure 6:**
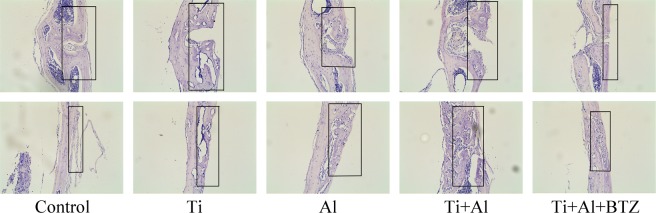
Histological staining of calvaria sections. Representative hematoxylin and eosin (H&E) stained histological slices. Severe calvarial destruction was found in Ti particles treated group. Al-NPs and BTZ significantly reduced the Ti particle-induced calvarial destruction. Rectangle frame indicated the surgical areas which the bone resorption was induce in the experimental groups. (Bar = 200 μm).

**Figure 7:**
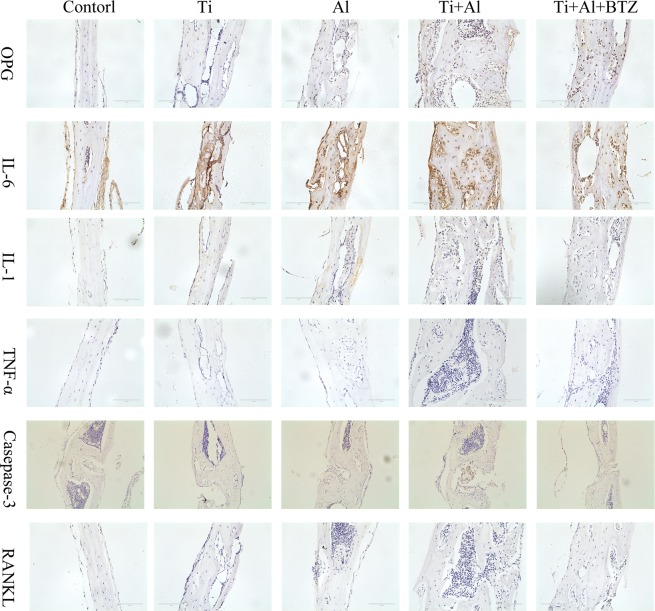
Immunohistochemical stains slices. Effect of Al-NPs and/or BTZ on inflammatory response (IL-1, IL-6, TNF-α),RANKL,OPG and casepase-3 in Ti particles-induced calvarial osteolysis model. (Bar = 100 μm).

Histological assessment confirmed that Ti particles implantation resulted in severely bone destruction and osteolysis. Histological assessment and histomorphometric analysis further confirmed that Al-NPs and BTZ treatment attenuates particles-induced bone erosion *in vivo* (Fig. 6). To further confirm the effect of Al-NPs and BTZ on particle disease induced by Ti particles, Immunohistochemistry assay was conducted to evaluate inflammatory and autophagy related cytokines. Immunohistochemical stains showed that an intense inflammatory infiltration in Ti implantation group, including TNF-α, IL1, IL-6, while Al-NPs and BTZ reduced inflammatory infiltration. What’s more, Al-NPs or BTZ caused the high expression level of OPG which was vital for bone reconstruction (Fig. 7).

## Discussion

Ti has been widely used as one of biomedical implant materials. During loading wear, Ti particles may inevitably be released from the surface of implants and influence the cell behavior of peri-implant osteoblasts. In this study, we found that Al-NPs and BTZ suppresses the activation NF-κB and evoked the autophagy process, which was supported by *in vitro* and *in vivo* assays. The effect of BTZ on the NF-κB signaling pathway is consistent with our previous studies^38^.

Metal biomaterial is commonly used worldwide as the biomedical implant materials for treating severe forms of skeletal disease or bone defect. Previous literatures had showed that wear particles generated during the daily function of implants is an inevitable consequence, which finally are believed that they will cause inflammatory osteolysis and particle disease. Although the development of materials (Metal, Polyethylene, Polymethylmethacrylate, and Ceramic) undergoes a marvelous evolution, yet none can be considered to be absolutely perfect. Nowadays, alloy is used for its superior corrosion resistance, and different types of bearing surfaces are available^49,50^. Ti particles released from Ti bulk implant or composites was confirmed by studies, and it was inevitable to apply Ti as a biomaterial, considering its outstanding mechanical property^1,2,13,14,16,17,22^. However, little is known about the interaction existing in different materials^50^.

What is the effect of particle amount on the cell? Actually, when we developed this project and other projects related to particle disease, this was the first question we cared and had to answer. So, we tested the apoptosis assay of MG-63 cells induced by Ti-μ particles. As the Fig. 1 shown, when combined Ti-μ particle with Al-n particle in the 5 μg/ml Ti+ 5 μg/ml Al group, the apoptosis of MG-63 cells deceased compared to 10 μg/ml Ti. There were two possible reasons for these results. The first possible reason was that the amount of particles had a more significant effect on the apoptosis of MG-63 in the mixed groups. This is not true. When Ti-μ increased to 10 μg/ml in the 10 μg/ml Ti+ 10 μg/ml Al group, in which the total concentration of particle was 20 μg/ml, it was interesting to find that the apoptosis of MG-63 did not increase to a level higher than the 10 μg/ml Ti group. In an opposite direction, the apoptosis of MG63 was lower in the 10 μg/ml Ti+ 10 μg/ml Al group compared with the 10 μg/ml Ti group. The second possible reason was that the Al-n could reduce the toxicity of Ti-μ but not the amount of particle. When we kept the same concentration of particle in the 5ug/ml Ti+ 5 ug/ml Al group, 10 μg/ml, the apoptosis of MG63 was reduced compared to the 10 μg/ml Ti group. So, we focused on the effect of Al-n reducing the toxicity of Ti-μ in the following assay.

Ti particles could activate NF-κB signaling pathway (Fig. 3A) and increased inflammatory mediators, such as IL-1β, IL-6, TNF-α (Fig. 4A–C). We found that co-cultured Ti with Al-NPs could slightly inhibit the degradation of the NF-kB inhibitory subunit IκBα and suppressed the activation NF-kB. What’ more, it lightly reduced the generation of inflammatory mediators^41,51^. And with our further study, we found that the Al signifiganct prevented the IL-1β expression (^#^p < 0.05) via attenuating the NF-kB signaling activator β-TRCP and reducing the expression of Casepase-3 (Fig. 5). Previous studies believed that aluminum is closely related to inflammation occurs in brain and mucosal^52^. According to the results obtained from this study, Al particles slightly inhibited the osteolysis, and co-culture of aluminum particles did not aggravate the local inflammatory environment. The results may be contradict, because the concentration of particles used is different. Meanwhile, the efficiency of Al particles is limited, so it cannot used as a therapeutic agent alone unless further literature to prove its validity, but which offered a basic data for implant design while it was inevitable to apply Ti as a biomaterial considering its outstanding mechanical property. Previous studies indicated that peri-implant osteolysis cannot be control even when osteoclast activity was inhibited, which suggest osteoblast cells may play a vital role in peri-implant osteolysis. Bone remodeling and homeostasis rely on the balance of osteogenesis and osteolysis. In the same concentration (10 μg/ml), Ti was more toxic than Al-NPs. Based on these results, in order to minimize the effect of concentration, figure out the effect of Al-NPs on Ti particle and further understand how did wear particles modulate osteoblastic activity, we examined the response of MG63 osteoblast-like cells exposed to Ti particle with or without Al-NPs. Ti particles induced apoptosis and necrosis in a dose-dependent manner in MG-63 cells (Fig. 1A–C).

Bortezomib (BTZ), a proteasome inhibitor is at the first considered as the frontline treatment of multiple myeloma, and it is now also considered by previous studies as a remedy for inflammatory response^44–46,53^. BTZ depress the secretion of inflammatory media by blocking the degradation of kappa B and thereby inhibiting the transfer of NF-κB to the nucleus^39^. Our previous study showed that BTZ prevent the inflammation of periodontal ligament cell without influencing cell activity or cell circle^45^. p50-p65-IκBα always presented an inactive form in the cytoplasm in the absence of stimulation. External stimuli such as rankle and TNF-α can activate NF-κB signaling pathways. Consequently, IκBα is ubiquitinated, phosphorylated, and then degraded via the ubiquitin proteasome system (UPS). Ultimately, NF-κB is translocated into the nucleus and enhanced the transcription of pro-inflammatory genes. In our study, levels of IκBα and p-IκBα in MG-63 cells were detected at the protein level with WB. IκBα decreased after Ti particles treatment for 3 days (Fig. 3A). Nevertheless, a mixture of titanium and aluminum group had a lower level of p-IκBα/IκBα than in Ti particles group. Furthermore, BTZ treatment showed a decrease in the accumulation of p-IκBα/IκBα in MG-63 cells. This study showed that BTZ could block the degradation of IκBα and inhibit the activation of the NF-κB induced by Ti particles in MG-63 cells. The presence of titanium particles in the peri-implant region can be an obstacle for bone regeneration. At the same time BTZ has also been shown to induce NF-κB pathways in immune cells in cancer immunotherapy when used for cancer combinatorial approach, suggesting that the effect of BTZ may be cell-specific^54^. Further research is needed if we are to make further clinical use.

The autophagy can be a protective mechanism to prevent cell from apoptosis, and the relationship between NF-κB signaling pathway and autophagy is still specific^55,56^. Our results showed that the expression of LC3 increased in the western blot assay (Fig. 3A) and immunofluorescence staining assays in Ti group in MG 63 cells and this high expression in Al-NPs group (Fig. 3B), and these results agreed with apoptosis and necrosis assay and the PCR test of the casepase-3 expression. But the casepase-3 on m-RNA level in Ti particle group could be blocked by Al-NPs but not by BTZ which indicated that there should be other signaling regulated the biological effect of Al-NPs on Ti particles besides NF-κB signaling path way (Fig. 4D). And then, our study showed that Al-NPs prevented the toxicity of Ti particles in MG63 cell via evoking the expression of LC3 and the autophagy process and blocking the apoptosis pathway. These result proposed LC3 as a critical bio-parameter to predict the quality of osteoblast exposed to Ti particles.

In the homeostasis of bone resorption and bone formation, bone mass is preserved. Aseptic loosening is associated with a serious imbalance of bone metabolism. This study has demonstrated the effect of Al-NPs and BTZ on Ti-particle-induced osteolysis using the murine calvarial model. We could see that Al-NPs and BTZ alleviated the bone destruction of murine calvarial induced by Ti particles in hematoxylin and eosin (H&E) stained histological slices. Besides, we also notice that samples from Ti + Al group were much thicker than other groups, and we speculate if there was new bone formation around the bone destruction area (Fig. 6). The Immunohistochemical stains data also showed that the inflammatory factor, apoptosis and activation of bone resorption signaling in Ti group was blocked by co-treated by Al-NPs and/not BTZ and the bone formation signaling was evoked in Ti and Al-NPs mixing group and co-treated BTZ group (Fig. 7).

## Methods

### Cells, media and reagents

Human osteoblast-like MG-63 cells were purchased from Obio Technology (Shanghai, China) Corp, Ltd. The cells were plated in MEM medium (#41500–034, Gibco), containing 10% fetal bovine serum(Natocor, SFBE) and 1% penicillin/streptomycin and inoculated in 5%CO2 at 37 °C. Although MG63 cells were osteosarcoma derived, MG63 cells exist features similar to poorly differentiated osteoblasts or human osteoblast precursors. Specific antibodies against inhibitor of κBα (4814S), phospho- inhibitor of κBα (9246S), cleaved caspase-3 (9661S) were purchased from Cell Signaling Technology. TNF-α (ab6671) antibody were purchased from abcam. LC3 (L7543) antibody were purchased from Gibco. Glyceraldehyde-3-phosphate dehydrogenase GAPDH (BS-0978M), IL-6 (BS-6309R), IL-1 (BS-6319R), p65 (BSM-33117M), phospho-p65 (BS-3485R), OPG (BS-0431R) antibodies were obtained from Bioss. Bortezomib (PS-341) was obtained from Selleck Chemicals (Houston, TX, USA).

### Materials and sterilization

Commercial pure Titanium oxide particle (Ti, #224227, <5 μm) and Aluminum oxide, nanoparticles (Al-NPs, #544833, <50 nm) were purchased from SIGMA. Practices were sterilized using the gamma irradiation (≥25kGy, Zhongjin Irradiation Chengdu Co, Ltd.)^57,58^. For *in vitro* experiments, particles were suspended in phosphate-buffered saline (PBS) at a concentration of 1 mg/ml as stock solutions 4 °C. The particles were further diluted in cell culture medium to attain different concentrations ranging from 5 to 50 µg/ml before exposed to MG-63 cells *in vitro*.

### Apoptosis assay

The effect of Ti and Al-NPs on the apoptosis of MG-63 cells was determined by Apoptosis and Necrosis Assay (Beyotime Biotechnology, Shanghai, China). MG-63 cells (1 × 10^5^ cells/well) were seeded in 6-well plates and cultured overnight, then treated with different concentrations practices: 10 µg/ml Ti, 10 µg/ml Al-NPs, 5 µg/ml Ti+ 5 µg/ml Al-NPs, 10 µg/ml Ti+ 10 µg/ml Al-NPs, 50 µg/ml Ti, 50 µg/ml Al-NPs (Control, Ti 10, Al-NPs 10, Ti 5 + Al-NPs 5, Ti 10 + Al-NPs 10, Ti 50, Al-NPs 50) for 3 days. After exposed to particles for 3 days, cells apoptosis detection was done with the Apoptosis and Necrosis Assay Kit. In brief, cells were washed with PBS twice and trypsinized. Both cells and supernatant were harvested, washed twice and resuspended. Subsequently, cells were added with Hoechst (5ul) and PI (5ul) for 30 min in the dark. The effect of BTZ on the apoptotic of MG-63 cells was determined using Apoptosis and Necrosis as well. That is, MG-63 cells (1 × 10^5^ cells/well) seeded in 6-well plates were cultured overnight, then treated with different concentrations BTZ (0 nM, 0.5 nM, 1 nM, 5 nM, 10 nM, 50 nM, and 100 nM) for 3 days. Then, cells were assessed as previously described.

### MTT assay

To detect the toxicity and safety of BTZ on MG-63 cells, MG-63 cells (2000 cells/well) were plated in 96-well plates treated with BTZ at different doses (0 nM, 0.5 nM, 1 nM, 5 nM, 10 nM, 50 nM, and 100 nM). Subsequently, proliferation viability of MG-63 cells were tested using 3-(4, 5-dimethylthiazol-2-yl)-2, 5-diphenyl tetrazolium bromide (MTT) assay (Beyotime Biotechnology, Shanghai, China) according to instructions of manufacturer on day0, day1, day2, day3 after treating with BTZ. The optical density (OD) was measured by EnSpire 23001100 at 570 nm.

### Western blot analysis

MG-63 cells were seeded at 5 × 10^5^ cells into 10 cm plates and treated with 10 µg/ml Ti, 10 µg/ml Al-NPs, 5 µg/ml Ti+ 5 µg/ml Al-NPs, 5 µg/ml Ti+ 5 µg/ml Al-NPs + 0.5 nM BTZ (Control, Ti 10, Al-NPs 10, Ti 5 + Al 5, Ti 5 + Al-NPs 5 + BTZ). Cells were harvested after treating with particles for 3 days and lysed. Centrifuged at 11,000 g for 15 min and extracted the supernatant. An enhanced BCA protein assay kit was applied to measure the protein concentration. Then mixed with loading buffer and boiled for 10 min. 30 µg total protein was separated by sodium dodecyl sulfate–polyacrylamide gel electrophoresis and were shifted onto the polyvinylidene-difluoride (PVDF) membrane. Subsequently, membranes were incubated with primary antibodies against GAPDH, p65, phospho-p65, inhibitor of κBα, phospho- inhibitor of κBα, LC3, cleaved caspase-3 overnight at 4 °C after blocking with 5% bovine serum albumin (BSA) or Skim milk for 1–2 h. Then the membranes were reacted with anti- mouse or anti- rabbit secondary antibody respectively for 1 h at room temperature, then visualized with an enhanced chemiluminescence (ECL) detection kit (Thermo, 34096).

In order to further detect the effect of Ti and Al-NPs on the activity of NF-ƙB signaling, MG63 cells were seeded and then treated with 10 µg/ml Ti, 10 µg/ml Al-NPs, 5 µg/ml Ti+ 5 µg/ml Al-NPs, 10 µg/ml Ti+ 10 µg/ml Al-NPs, 50 µg/ml Ti, 50 µg/ml Al-NPs, (Control, Ti 10, Al-NPs 10, Ti 5 + Al-NPs 5, Ti 10 + Al-NPs 10, Ti 50, Al-NPs 50,Ti 10 + Al-NPs 10 + BTZ(0.5 nM)), and the protein expression of β-TRCP was analysis as described above.

### Quantitative polymerase chain reaction (PCR) analysis

For real-time PCR, 1 × 10^5^ MG-63 cells were seeded in a 6-well plate. Cells were then treated with 10 µg/ml Ti, 10 µg/ml Al-NPs, 5 µg/ml Ti+ 5 µg/ml Al-NPs, 5 µg/ml Al-NPs + 5 µg/ml Al-NPs + 0.5 nM BTZ (Control, Ti 10, Al-NPs 10, Ti 5 + Al-NPs 5, Ti 5 + Al-NPs 5 + BTZ) for three days, respectively. According to the manufacturer’s instructions, total RNA was prepared using TRIzol reagent (15596026, Invitrogen) and reverse to cDNA using reverse transcriptase. Real-time PCR was performed with the SYBR Premix Ex Tag kit (RR820A, Takara) by CFXConnect. The PCR conditions of this detector was as following: 95 °C for 2 min, 40 cycles of denaturation at 95 °C for 5 s and amplification at 60 °C for 30 s. Relative mRNA expression was analyzed using the 2−ΔΔCt relative expression method. Primers sequences were shown in the Fig. 4E.

### Enzyme-linked immunosorbent assay (ELISA)

Protein expression was determined using Enzyme-Linked Immunosorbent Assay (ELISA). 1 × 105 MG-63 cells were seeded in a 6-well plate. Cells were then treated with 10 µg/ml Ti, 10 µg/ml Al-NPs, 5 µg/ml Ti+ 5 µg/ml Al-NPs, 10 µg/ml Ti+ 10 µg/ml Al-NPs, 50 µg/ml Ti, 50 µg/ml Al-NPs, (Control, Ti 10, Al-NPs 10, Ti 5 + Al-NPs 5, Ti 10 + Al-NPs 10, Ti 50, Al-NPs 50) for three days, respectively. And the concentration of IL-1β was measured with ELISA kits (Bioss, bsk11001) according to the manufacturer’s instructions, compared with the control group. (^#^p < 0.05).

### Ti and Al-NPs-induced mouse calvarial osteolysis model

We established a mouse calvarial osteolysis model to determine the preventative effects of Al and BTZ on osteolysis induced by Ti particles *in vivo* as previously reported^31,32,59^. Animal experiments were performed according to the principle of the National Institutes of Health (NIH) Guide. All experiments were approved by the Ethical Committee for Animal Experimentation of Chongqing Medical University. Twenty-five C57BL/6J male mice, aged 6–8 weeks, were purchased from the Laboratory Animal Research Center of Chongqing Medical University. The calvarial osteolysis model of Ti and Al-NPs induced was established as previously described^35,41^. Mice were randomly assigned to five groups (5 animals per group): Control, Ti (10 mg dried Ti particles), Al-NPs (10 mg dried Al-NPs), Ti+ Al-NPs (5 mg dried Ti particles + 5 mg dried Al-NPs) and Ti + Al-NPs + BTZ (5 mg dried Ti particles + 5 mg dried Al-NPs + BTZ). Briefly, the mice were anesthetized by intraperitoneal injection. The hair was shaved carefully and sterilized with disinfectant (Iodoform and 70 alcohol). A 1 cm length incision along the mid-line was made, and periosteum over the calvarium was exposed with the periosteal elevator and hemostatic forceps. Then, the periosteum of the calvarium was scraped to generate a 1 cm × 1 cm area around the mid-line crossing the front and bregma bone in the cranium of mouse. The control group directly stitched the incision without further embedded particles. In the Ti group, Ti particles (10 mg) were embedded around the middle suture. In the Al-NPs group, Al-NPs (10 mg) were embedded around the middle suture. In the Ti + Al-NPs and Ti + Al-NPs + BTZ groups, Ti particles (5 mg) and Al-NPs particles (5 mg) were embedded around the middle suture. Practices were uniformly spread over the periosteum. All of the surgical instruments have been sterilized by high temperature and high pressure. All surgery operations were finished on the same day. Two days after inserting particles, BTZ was injected into the periosteum in the Ti + Al-NPs + BTZ group every other day locally. After 14 days, Animals were sacrificed by cervical dislocation. The calvarial caps were obtained by segregating the bone free with the scalp and underlying brain tissue. And the calvariae were fixed in 4% para-formaldehyde for histological experiment, as well as Immunohistochemistry assay.

### Histology and bone histomorphometry analysis

Harvested calvariae were fixed with 4% paraformaldehyde for 24h–48h, and decalcified in 10% EDTA for 1 month, followed by embedded in paraffin. 5 μm thick sections of the calvaria were taken in the coronal plane in the operation area and stained with hematoxylin and eosin (H&E) for histological analysis. Then, images were collected by a high-quality light microscopy. The skull thickness and the eroded surface was observed and quantified to determine the inflammatory cell infiltration^60^.

### Immunohistological analysis

Histological sections were prepared following the product manual (pv-9001, ZSJB-BIO, China), In a nutshell, sections cultured with antigen-retrieval buffer, blocked with 5% hydrogen peroxidase for 5 min, and incubated with primary antibodies, such as receptor activator of NF-κB ligand (RANKL), OPG, IL-6, IL-1, TNF-α and caspase-3 over-night at 4 C. The sections were washed, then incubated with biotin-conjugated secondary antibody for 30 min. With counterstaining by hematoxylin, the color of sections presented using 3.3 0 -diaminobenzidine tetrahydrochloride.

### Statistical analysis

Data was presented as means ± standard deviation (SD). Statistical analysis between groups was performed with one-way analysis of variance (ANOVA). A value of p < 0.05 (*) was considered statistically significant.

## Supplementary Information

Supplementary Information.
