# Supplementary material for: Nanosized Alumina Particle and Proteasome Inhibitor Bortezomib Prevented inflammation and Osteolysis Induced by Titanium Particle via Autophagy and NF-κB Signaling
Source: Sci Rep. 2020 Mar 27;10:5562. doi: 10.1038/s41598-020-62254-x (PMC7101404; doi:10.1038/s41598-020-62254-x)
Supplement: Supplementary file 1 — Supplementary Information. [file 41598_2020_62254_MOESM1_ESM.doc]

**Nanosized Alumina Particle and Proteasome Inhibitor Bortezomib Prevented inflammation and Osteolysis Induced by Titanium Particle via Autophagy and NF-κB Signaling**

**Zhiwei Zhang** ^1,2,4^,**^+^**, **Xuewei Fu** ^1,2,5^,**^+^**, **Ling Xu** ^1,2,5^, **Xiaolei Hu** ^6^, **Feng Deng** ^1,2,3^, **Zhiqiang** **Yang** ^1,2,7^, **Lin Jiang** ^1,2^, **Tiwei Fu** ^1,2^, **Pengfei Zhou** ^1,2^, **Jinlin Song** ^1,2,3^, **Ping Ji** ^1,2,7^, **Jiao Huang**^1,2,4^,**^*^** , **Xiaomian Wu** ^1,2,3^,**^*^**

**+ These authors contributed equally to this work.**


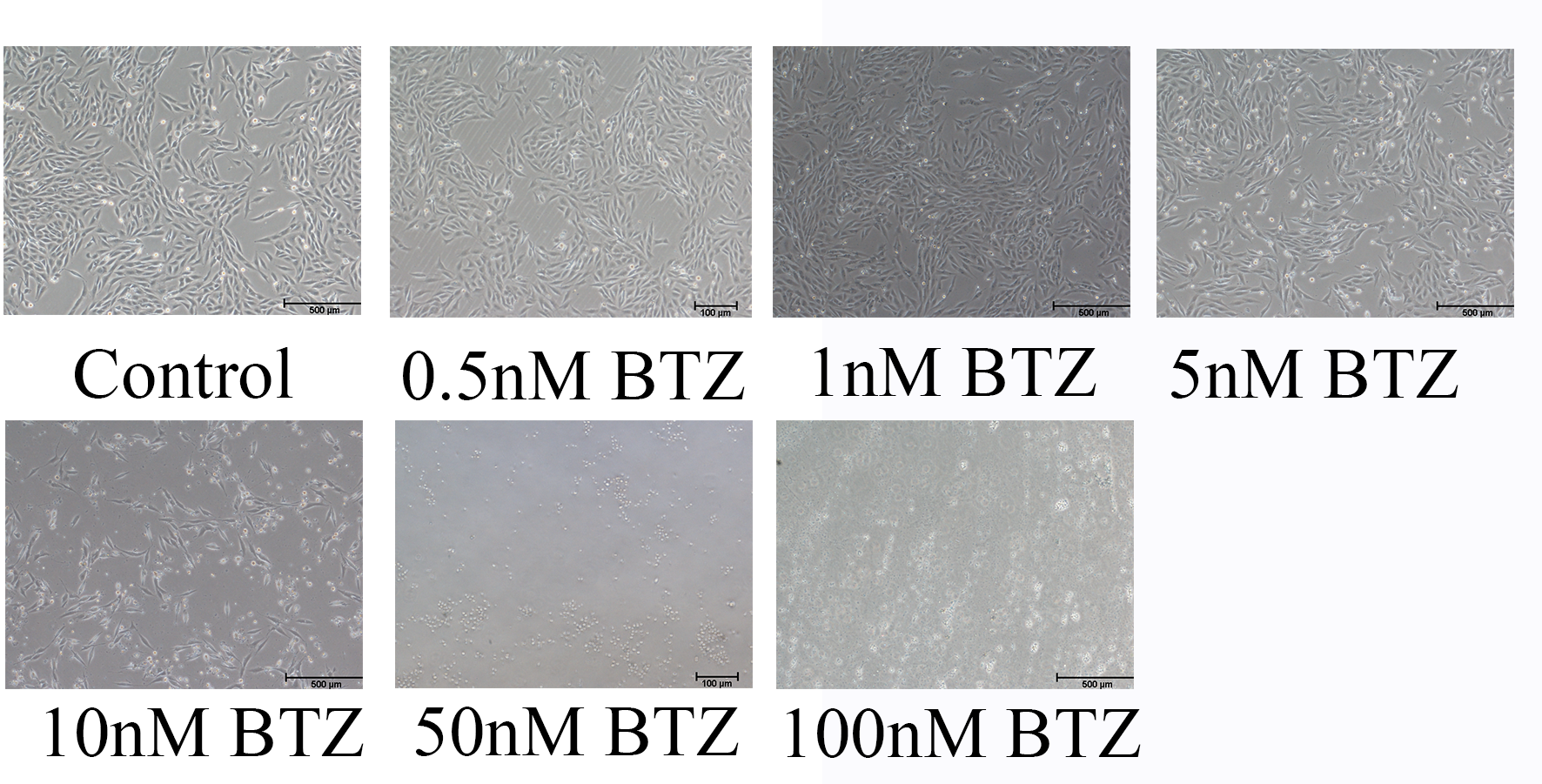


**Supplementary Figure 1.** Morphology of MG63 cell exposed to different concentration of Bortezomib for 24 hours.


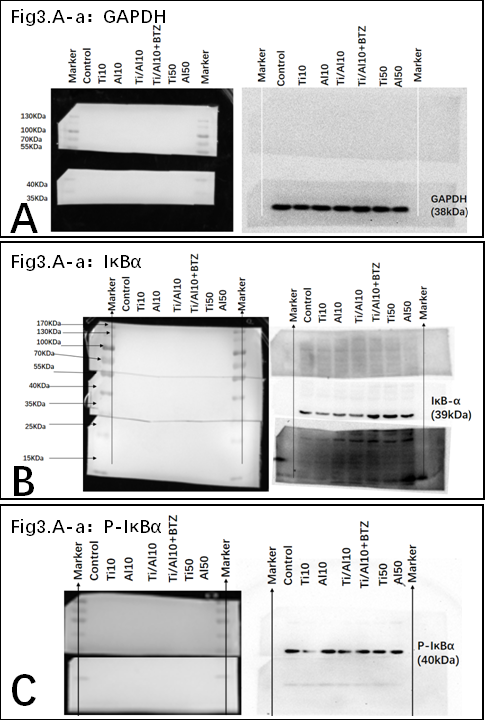


**Supplementary Figure 2. The original data of main figure 3.A-a. :**The full-length gels and blots showed that the GAPDH (A), IƙBɑ(B), and P-IƙBɑ(C) were detected at 38kDa,39kDa and 40kDa respectively.


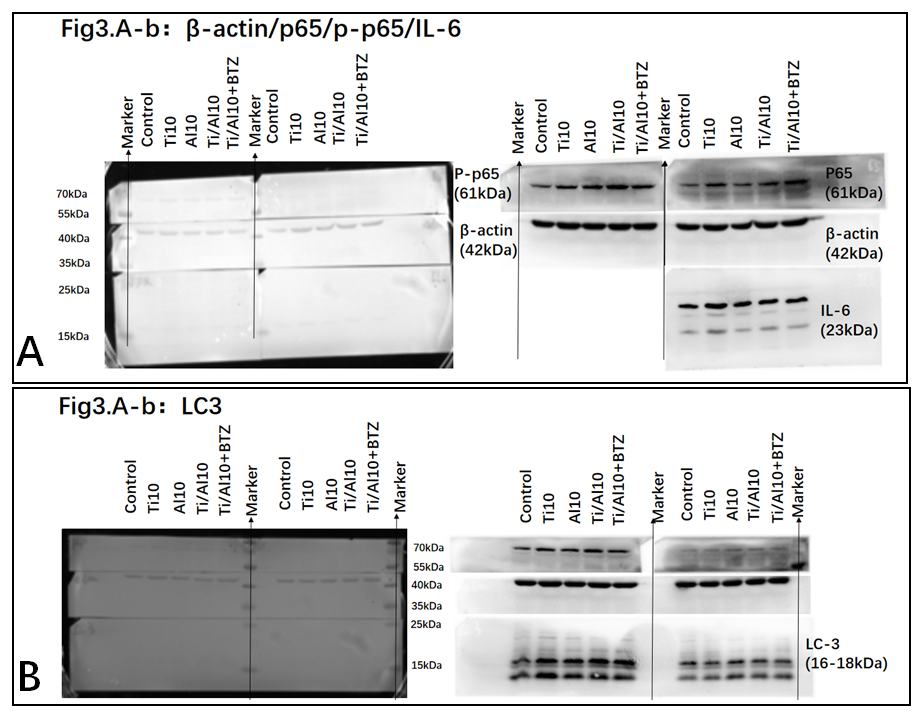


**Supplementary Figure 3. The original data of main figure 3.A-b.:**The full-length gels and blots showed that the β-actin (A), P65 (A), P-p65 (A), IL-6 (A) and LC-3(B) were detected at 42kDa, 61kDa,61kDa,23kDa and 16-18kDa respectively.


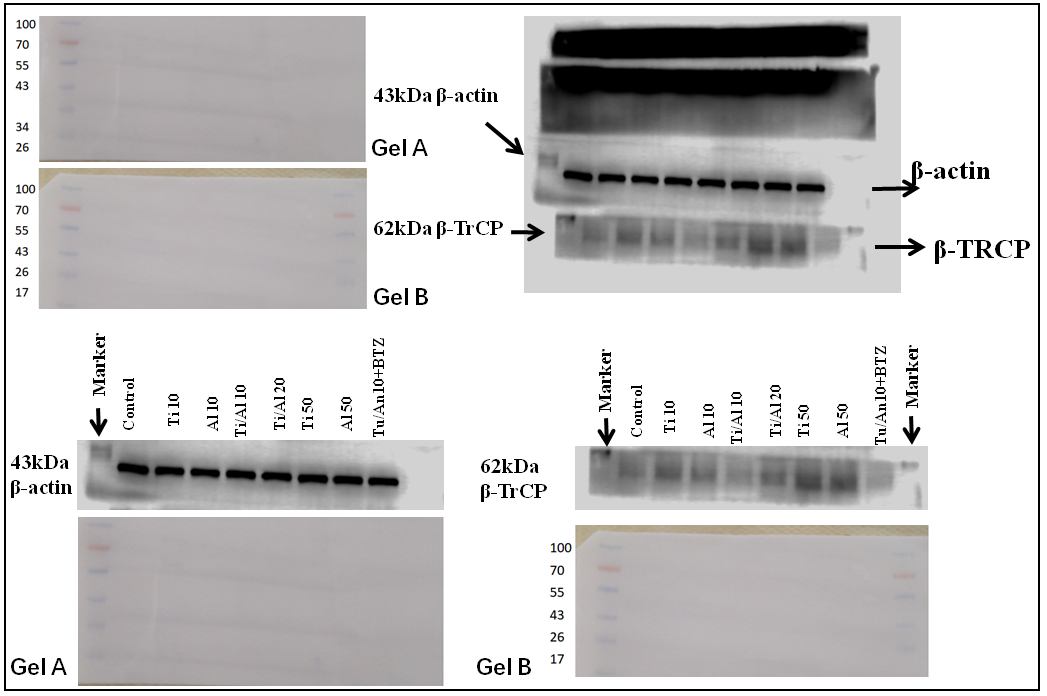


**Supplementary Figure 4. The original data of main figure 5.** The full-length gels and blots showed that the β-TrCP and β-actin was detected at 62 kDa and 43 kDa respectively. （β-actin was developed from Gel A and β-TrCP was developed from Gel B）
